# Use of Principal Components Analysis and Kriging to Predict Groundwater-Sourced Rural Drinking Water Quality in Saskatchewan

**DOI:** 10.3390/ijerph14091065

**Published:** 2017-09-15

**Authors:** Lianne McLeod, Lalita Bharadwaj, Tasha Epp, Cheryl L. Waldner

**Affiliations:** 1 Western College of Veterinary Medicine, University of Saskatchewan, 52 Campus Drive, Saskatoon, SK S7N 5B4, Canada; lianne.mcleod@usask.ca (L.M.); tasha.epp@usask.ca (T.E.); 2 School of Public Health, University of Saskatchewan, 104 Clinic Place Saskatoon, SK S7N 2Z4, Canada; lalita.bharadwaj@usask.ca

**Keywords:** exposure assessment, groundwater, water quality, arsenic, principal components analysis, kriging, geostatistics, Saskatchewan

## Abstract

Groundwater drinking water supply surveillance data were accessed to summarize water quality delivered as public and private water supplies in southern Saskatchewan as part of an exposure assessment for epidemiologic analyses of associations between water quality and type 2 diabetes or cardiovascular disease. Arsenic in drinking water has been linked to a variety of chronic diseases and previous studies have identified multiple wells with arsenic above the drinking water standard of 0.01 mg/L; therefore, arsenic concentrations were of specific interest. Principal components analysis was applied to obtain principal component (PC) scores to summarize mixtures of correlated parameters identified as health standards and those identified as aesthetic objectives in the Saskatchewan Drinking Water Quality Standards and Objective. Ordinary, universal, and empirical Bayesian kriging were used to interpolate arsenic concentrations and PC scores in southern Saskatchewan, and the results were compared. Empirical Bayesian kriging performed best across all analyses, based on having the greatest number of variables for which the root mean square error was lowest. While all of the kriging methods appeared to underestimate high values of arsenic and PC scores, empirical Bayesian kriging was chosen to summarize large scale geographic trends in groundwater-sourced drinking water quality and assess exposure to mixtures of trace metals and ions.

## 1. Introduction

Several studies have highlighted associations between exposure to arsenic from drinking water and a variety of chronic diseases including type 2 diabetes, hypertension, cardiovascular disease and cancer [1,2,3,4,5,6,7]. Poor water quality, especially aesthetic issues that impact consumer acceptance of tap water, might also have indirect impacts on the development of chronic diseases such as diabetes and cardiovascular disease by motivating the consumption of sugar-sweetened beverages as an alternative to drinking water [8]. Additionally, mixtures of contaminants may act synergistically and have impacts that are not appreciated when examining the isolated effects of individual contaminants [9,10,11].

In Canada, drinking water oversight is a provincial mandate. Guidelines for acceptable levels of contaminants and regulations pertaining to water quality testing are established by each province, but typically follow recommendations set forth in the federal Guidelines for Canadian Drinking Water Quality [12,13]. In the province of Saskatchewan (SK), the Drinking Water Quality Standards and Objectives [14] apply to regulated water supplies. Standards are legally enforceable and are based on parameters that are potentially harmful to human health. Objectives are guidelines based on parameters that are not considered harmful to human health in the context of expected concentrations in drinking water, but which can negatively impact the aesthetic qualities of the water.

In Saskatchewan, three tiers of drinking water systems are defined, of which two are subject to regulation [15]. Public drinking water systems are monitored and regulated by the Saskatchewan Water Security Agency and semi-public supplies are monitored by the Ministry of Health. Private water supplies serve a single household or farm; these supplies are not regulated and have no monitoring requirements. Residents who use private water supplies may lack the knowledge and resources to routinely and adequately test their household water source, and therefore could be at greater risk of exposure to drinking water of poor quality or that has elevated concentrations of contaminants such as arsenic. Similarly, smaller water distribution systems may lack the resources to avoid placing wells in aquifers of lower water quality, and the requirements for testing are based on the size of the population served with less stringent requirements for smaller supplies [16]. Therefore, residents in rural areas, whether using a smaller public system or a private water supply, may be vulnerable to health effects related to the quality of water available at their household tap. Access to affordable tap water alternatives such as bottled water may also be limited in rural areas as compared to those available in urban locations, exacerbating the potential impacts of poor tap water quality for those located outside urban centers.

Ground water quality has been previously identified as a public health concern in SK, particularly for residents using private wells for their drinking water [17,18,19,20,21]. Approximately 28% of SK residents, primarily in rural areas, use groundwater-based public drinking water supplies and another 15% obtain drinking water from private water supplies [22]. A small study of arsenic levels in 61 private and rural municipality-owned wells in SK found that 23% of the wells had arsenic concentrations above the current SK standard of 0.01 mg/L [17]. Another study found that 99.6% of 535 wells sampled exceeded at least one aesthetic objective or health standard and, of those, 35% exceeded a health-related standard [23]. In addition, other minerals and ions such as iron and manganese, though not considered substantial health risks, are frequently found in groundwater at concentrations that exceed recommended levels and negatively affect the aesthetic quality of drinking water [19,23]. In a recent survey of residents in rural SK, 25% of respondents reported having an aesthetic complaint about their household drinking water [24].

Our goal was to summarize the quality of ground-sourced drinking water as part of the exposure assessment for the investigation of associations between tap water quality and rates of type 2 diabetes and cardiovascular disease in rural SK. Estimating exposure to constituents of drinking water is a major challenge in investigating such associations. Previous work has suggested that geostatistics can be used to map estimated exposure to arsenic through groundwater as a continuous surface across a region based on a limited set of point measures [25,26,27,28,29]. Additionally, principal components analysis (PCA) has been used as a variable reduction method to investigate groupings of groundwater parameters [30,31,32,33] and has been combined with geostatistical methods to map underlying latent processes contributing to overall water quality across a region [34,35,36,37]. However, we are not aware of previous studies exploring the use of PCA and geostatistics to summarize groundwater quality in SK. We are also not aware of studies exploring the use of PCA to summarize mixtures of contaminants for exposure assessment in epidemiologic studies.

Previously collected water surveillance records represent an existing source of data by which water quality can be summarized as a means of exposure assessment for epidemiological models of associations between household water quality and health. Our objective was to evaluate the use of existing water quality monitoring data from public systems and private water supplies to describe ground-sourced drinking water quality, and use PCA to summarize metals and ions included in the monitoring of health standards and aesthetic objectives. Our secondary objectives were to compare the performance of ordinary kriging, universal kriging, and empirical Bayesian kriging for predicting arsenic concentrations and principal component scores derived from principal components analysis, and to evaluate the potential use of these tools in human health exposure analysis for large rural areas of western Canada. The results of this analysis were later used to estimate groundwater-sourced drinking water arsenic concentrations and principal components summarizing water health standard and aesthetic parameters for geographic regions across rural Saskatchewan. The estimates were needed to inform an exposure analysis for epidemiological investigation of associations between water quality and diabetes, hypertension, and cardiovascular disease in rural SK.

## 2. Materials and Methods

### 2.1. Study Area

**Figure 1:**
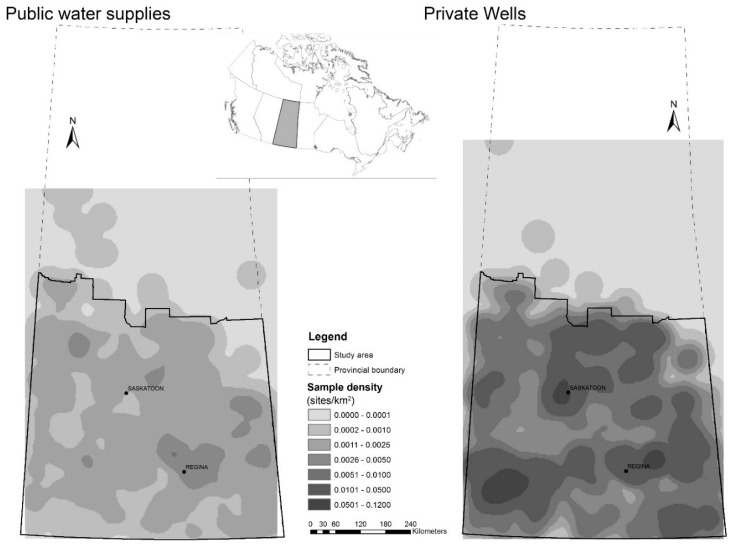
The density of sampling sites for: public water supplies (**left**); and private wells (**right**) across the province. The solid line indicates the boundary for the geostatistical analysis. Inset map shows location of the province of Saskatchewan within Canada.

Saskatchewan is a province located in central Canada with borders approximately located along 49° N and 60° N, and 102° W and 110° W. The geostatistical analysis was limited to the southern part of the province, because the water monitoring data were very sparse in the sparsely-populated northern part of the province (Figure 1). The study area for the geostatistical analysis was bounded by the solid line in Figure 1 and encompassed an area just over 327,900 km^2^ and was home to approximately 1 million people in 2010.

### 2.2. Data Sources

All data were obtained from the Saskatchewan Water Security Agency. The public supply data originated from 492 groundwater sourced systems that fall under regulation of the Water Security Agency and were collected during 1985–2012, and consisted primarily of treated water samples. Wells considered to be under the influence of surface water were excluded, but otherwise well depths were not readily available. The private water supply data were collected from 4093 wells as part of the Water Security Agency’s Rural Water Quality Advisory Program (RWQAP), a unique program designed to provide advice and subsidized water testing services to private well owners during 1996–2011. Well depth was reported by landowners, and subject to considerable uncertainty. Furthermore, well depth was not available for over 25% of samples; therefore, well depth was not considered in the analysis. All chemical analyses were performed using methods accredited by the Canadian Association for Laboratory Accreditation Inc. [38] at the Saskatchewan Disease Control Laboratory (Regina, SK, Canada) or the Saskatchewan Research Council Environmental Analytical Laboratories (Saskatoon, SK, Canada). The densities of sampling sites for public water supplies and private wells are shown in Figure 1.

All analyses were carried out separately for public water supply data and private well data. Concentrations for all parameters, for both private and public supply databases, were converted to mg/L. Only samples from groundwater sources were retained, and samples with missing location information were discarded. Because analytic methods and detection limits changed over time, data were compared to the detections limits corresponding to the sampling date; for samples where the parameter concentration was reported as below the detection limit, half the detection limit was imputed for the missing value [26,39]. The distributions for all parameters were right skewed and natural logarithm transformations were applied prior to analysis. Descriptive statistics for parameters of interest, along with the proportion of samples exceeding Saskatchewan’s standards or objectives, and the proportion of samples that were below detection limits were summarized for the public water supply data and the private well data.

Cyanide, mercury, cadmium, chromium, and fluoride were excluded from the analysis for both public supplies and private wells to maintain consistency in the analysis of the different types of supplies. Cyanide and mercury were sampled less frequently than the other metals and ions of interest in public systems. Cyanide was not sampled in the private wells and mercury was sampled infrequently. Additionally, 98% of cadmium and 72% of chromium samples were below detection limit for the public water supply data, and 92% of cadmium and 91% of chromium samples were below detection limit in the private well data. Fluoride was infrequently reported for private wells, but was added to some municipal supplies [40] and was also excluded.

The water quality data for the public water supplies consisted of repeated measures taken at irregular intervals during 1985–2012. Because kriging methods implemented in ArcGIS require a single measure at each point location, a generalized linear mixed model (GLMM) with no fixed effects was used to estimate a single predicted value for each water parameter for each water supply location, using the Mixed procedure with maximum likelihood estimation in SAS (SAS Institute Inc., Cary, NC, USA). The GLMM included a random intercept μ~N(0, σ^2^) to account for between supply variation, as well as a structured error term with a spatial power covariance structure based on the time between sampling to account for the non-equidistant repeated sampling within water supplies [41,42].

The private well data consisted of measures at a single point in time for each location. However, due to privacy considerations, private well locations were generalized to the centroid of the section of land on which they were located before confidential data access was granted for this analysis. A section is approximately 1.6 km by 1.6 km and corresponds to a parcel of land described by the grid-based land description system used in western Canada [43]. Wells with duplicated generalized locations were manually jittered by alternately increasing or decreasing the latitude and longitude by increments of 10 m for each subsequent duplicate well location until no duplicate locations remained.

### 2.3. Principal Components Analysis

Principal components analysis (PCA) is a multivariate statistical technique that can be used to reduce the dimensionality of a dataset with numerous correlated variables and categorize variables into groups based on their covariance. The resultant independent principal components account for the variance in the observed data, except for that explained by components that are not retained. These independent principal components can then be used as predictor variables in subsequent analyses. PCA was performed using SAS (SAS Institute Inc., Cary, NC, USA) and applied separately to the public supply and private supply data, separated into groups of water parameters identified as health standards and as aesthetic objectives according to the Saskatchewan Drinking Water Quality Standards and Objectives. For the public water supply data, the single predicted value for each supply was used in PCA, while values for each individual well were used in the PCA of private well data.

Log transformed concentrations (mg/L) were converted to standardized z-scores prior to performing PCA to mitigate the effect of measurement scales and to ensure that highly variable parameters did not dominate the analysis [44,45]. Kaiser’s measure of sampling adequacy (KMO) [46] and Bartlett’s test of sphericity [47] were performed to assess the sampling adequacy and correlation of these data and therefore their suitability for PCA. In each analysis, principal components (PC) with an eigenvalue of greater than one were retained [48], and subject to varimax rotation to maximize the variation explained by each of the retained principal components and to obtain the final principal component loadings and coefficients. Principal component (PC) scores for each of the retained principal components were calculated for the public supplies and private wells for use in the geostatistical analysis.

### 2.4. Geostatistical Analysis

Kriging is a geostatistical method that incorporates spatial autocorrelation in continuous variables to interpolate values at locations at which they have not been measured [49]. The basis of kriging is the semivariogram model, which uses the semivariance between point measures to summarize the spatial relationships in variables. Ordinary kriging and universal kriging are methods for which estimation is based on weighted least squares and the assumption that the calculated semivariogram is the true model for the data. Ordinary kriging differs from universal kriging in that ordinary kriging assumes a constant unknown mean across a given area, while universal kriging assumes a constant trend in the data [49].

Empirical Bayesian kriging first became available in ArcGIS version 10.1, and is based on restricted maximum likelihood estimation [50]. Additionally, it allows for uncertainty in the semivariogram model by a process of data subsetting and simulation to estimate a range of semivariogram models. As a result, empirical Bayesian kriging requires fewer assumptions about the semivariogram model form, is considered accurate for moderately non-stationary data, and provides more accurate estimation of prediction standard errors than other kriging methods [50].

Principal component scores and log transformed arsenic concentrations were mapped in ArcMAP 10.3 (ArcGIS, ESRI, Redlands, CA, USA) to compare the performance of ordinary, universal and Empirical Bayesian Kriging for the prediction of arsenic concentrations, PC scores for health standards (PC_health_), and PC scores for aesthetic objectives (PC_aesthetic_). The analyses for each of these three types of data were performed separately for public water supply data and private well data. 

Variogram models for each variable were developed using SAS 9.4 software (SAS Institute Inc., Cary, NC, USA) [51]. First or second order large scale trends were identified in the data for each variable using regression analysis. Where trends were detected, the residuals from the regression models were used to develop variogram models. The average nearest neighbor distance for the sampling locations for each variable were calculated in ArcGIS 10.3 and set as the lag distance for the variograms, and half the study area extent divided by the lag distance was used to calculate the maximum number of lags, which limits the semivariogram lag to half the extent of the sampling area [51]. The fits of spherical, exponential, and Gaussian models were compared using the Akaike Information Criterion (AIC) for each variable in each of the public and private water supply datasets. The presence of anisotropy was evaluated visually by dividing the variogram into eight directions (22.5° angles); however, for the final analysis, an omnidirectional variogram was modeled.

ArcMAP 10.3 (ArcGIS, ESRI, Redlands, CA, USA) was used to perform ordinary, universal and empirical Bayesian kriging for each variable. The order of trend removal and lag distance were set based on the variogram models developed in SAS. For the public water supply data, the number of lags was set to the maximum number of lags calculated for the SAS variogram models, but for the private well data 100 lags were used for each kriging model because the calculated maximum number of lags for each parameter exceeded the maximum of 100 allowed in ArcMAP. Because the spherical form of the variogram model had the lowest AIC for all but two of the variogram models (PC4_aesthetic_ for public water supplies and PC3_health_ for private wells), the model form was specified as spherical for each variable for ordinary and universal kriging. Because empirical Bayesian kriging is less interactive and spherical models are not available, the settings for Bayesian Kriging were left at default values, such that subsets of 100 points and 100 simulations were modeled for each variable using a power-based model.

The root mean square prediction error (RMSE) in the kriging cross validation analysis performed in ArcMAP was used to compare the ability of ordinary, universal, and empirical Bayesian kriging to predict values for each parameter [52]. In cross validation, each point is sequentially omitted from the dataset and the remaining points used to predict a value for that point; the difference between the predicted and measured value is the prediction error. The method that resulted in the greatest number of parameters with the lowest RMSE was identified as the optimal kriging method for the water data.

## 3. Results

For both public water supply (Table 1) and private well data (Table 2), the medians of most parameters, with the exception of manganese, were below the values specified in Saskatchewan’s Drinking Water Quality Standards and Objectives. However, the 95th percentile value exceeded the standard or objective for several parameters including arsenic, uranium, iron, manganese, sodium, sulfate, alkalinity, hardness and total dissolved solids. When the values for public systems after repeated measures modeling (Table 3) were compared to the summary statistics for the raw data (Table 1), the median values were similar, but the maximums were smaller.

### 3.1. Principal Components Analysis

Bartlett’s test of sphericity was satisfied for each dataset indicating that there was sufficient correlation among the variables for PCA to be useful (*p* < 0.001 for each dataset). The KMO measure of sampling adequacy was above the recommended minimum value of 0.6 [46] for the data for aesthetic objectives for public water supplies (KMO = 0.63), for health standards in private wells (KMO = 0.62) and aesthetic objectives for private wells (KMO = 0.68). However, the KMO was 0.49 for the health standards in the public water supply data. Removing the parameter with the lowest individual measure of sampling adequacy (arsenic) did not substantially improve the KMO. Despite the low KMO, PCA was completed for this dataset to facilitate planned comparisons. 

For health standards, analysis of the public supply data yielded three PCs accounting for 63.7% of the variance, while the private supply analysis yielded three PCs that accounted for 67.6% of the variance (Table 4). The first health standards principal component (PC1_health_) was associated with nitrate and selenium for both types of supplies. However, PC1_health_ was more strongly associated with uranium in private wells than in public supplies, while PC1_health_ was more strongly associated with lead in public supplies than in private wells. The second PC_health_ exhibited opposite pattern, demonstrating a positive association with boron and a negative association with barium in public water supplies, and a negative association with boron and positive association with barium in private wells. The third PC_health_ was associated with arsenic for the public supplies, but only weakly associated with arsenic in private supplies. For public supplies, PC3_health_ was also weakly associated with uranium, but, in the private supplies, PC3_health_ was associated with lead. 

For the aesthetic objectives, PCA on the public supply data yielded four PCs explaining 77.8% of the variance, while the private supply data yielded only three PCs accounting for 70.3% of variance (Table 4). For both types of supplies, the first aesthetic objectives PC (PC1_aesthetic_) was associated most strongly with alkalinity, chloride, sodium, sulfate, and total dissolved solids. In addition, for both types of supplies, the second PC_aesthetic_ was characterized by strong associations with hardness and magnesium, and weak associations with sulfate and manganese. In addition, private supplies had a weak association of zinc with PC2_aesthetic_. The third PC_aesthetic_ for public and private supplies were associated primarily with iron and manganese, but, in private systems, PC3_aesthetic_ also exhibited a negative association with copper. Conversely, the PC4_aesthetic_ retained only from the public supply was associated with copper and zinc.

### 3.2. Geostatistical Analysis

Of the 492 groundwater-sourced public water supplies sampled, 480 fell within the study area outlined in Figure 1 and were used in the geostatistical analysis. Arsenic concentrations were available from all 480 public water supply locations within the study area. Health standards PC scores were available for 459 locations and aesthetic objectives for 435 locations.

Of the 4093 private wells sampled, 4084 fell within the study area and were used in the geostatistical analysis. Arsenic concentrations were available for 4073 private wells within the study area; health standards PC scores were available for 3970 private wells; and aesthetic objectives for 3999 private wells in the study area. 

The number of data points, lag distance, and number of lags used as inputs in ordinary and universal kriging are summarized in Table 5. Visual inspection of plots for anisotropy suggested the few observed differences were minor, thus isotropic models were assumed for all variables. 

The RMSE values from each of the kriging models for each of the variables demonstrate that, overall, the Bayesian kriging resulted in the lowest RMSE for nine variables, whereas ordinary kriging resulted in the lowest RMSE for six variables (Table 6).

**Figure A1:**
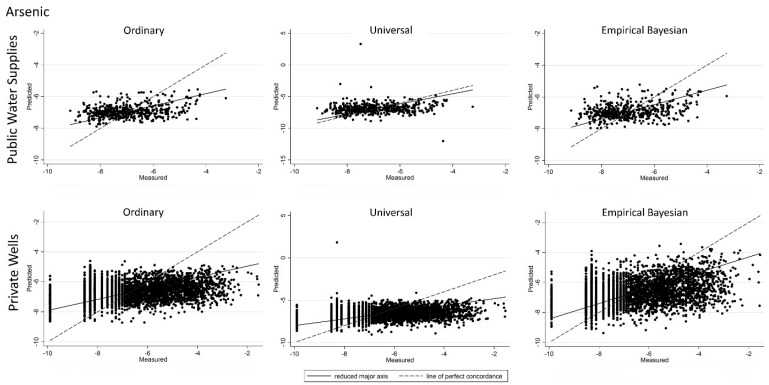
Predicted vs. measured values for the natural logarithm of arsenic concentration obtained from the cross-validation results for ordinary, universal, and empirical Bayesian kriging for: public water supplies (**top row**); and private wells (**bottom row**). Line of perfect concordance indicated by dashed line.

**Figure A2:**
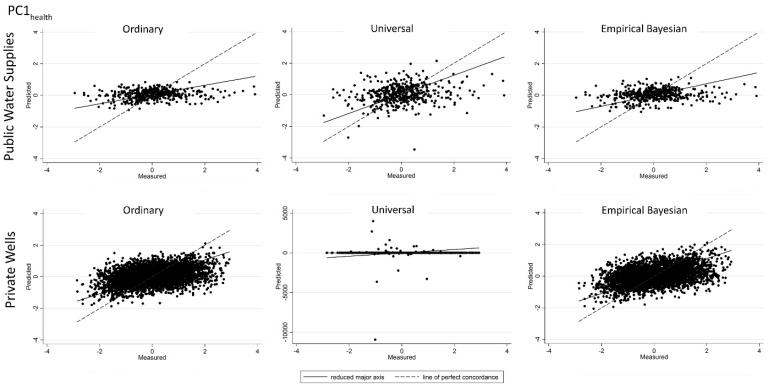
Predicted vs. measured values for PC1_health_ scores obtained from the cross-validation results for ordinary, universal, and empirical Bayesian kriging for: public water supplies (**top row**); and private wells (**bottom row**). Line of perfect concordance indicated by dashed line.

**Figure A3:**
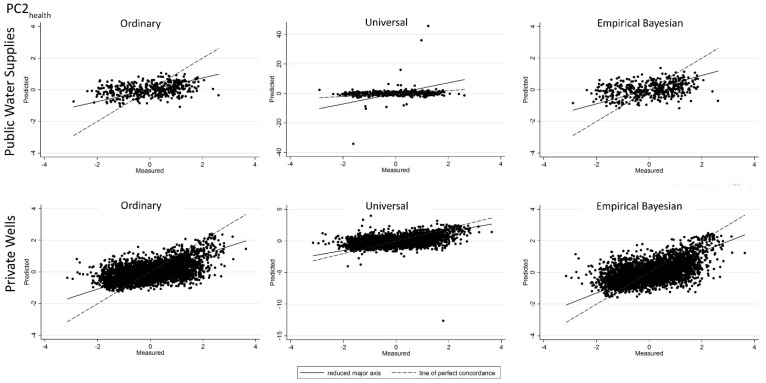
Predicted vs. measured values for PC2_health_ scores obtained from the cross-validation results for ordinary, universal, and empirical Bayesian kriging for: public water supplies (**top row**); and private wells (**bottom row**). Line of perfect concordance indicated by dashed line.

**Figure A4:**
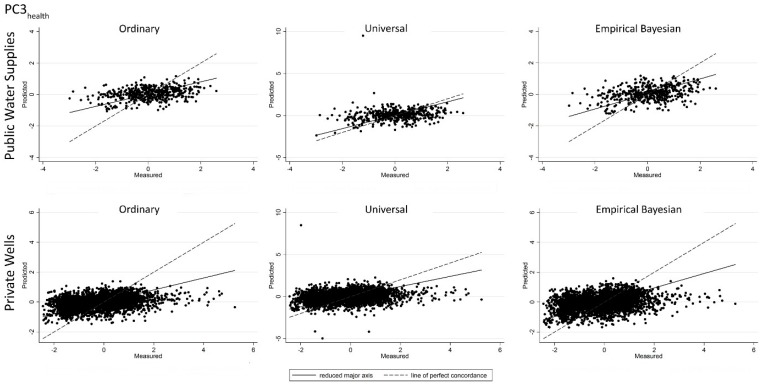
Predicted vs. measured values for PC3_health_ scores obtained from the cross-validation results for ordinary, universal, and empirical Bayesian kriging for: public water supplies (**top row**); and private wells (**bottom row**). Line of perfect concordance indicated by dashed line.

**Figure A5:**
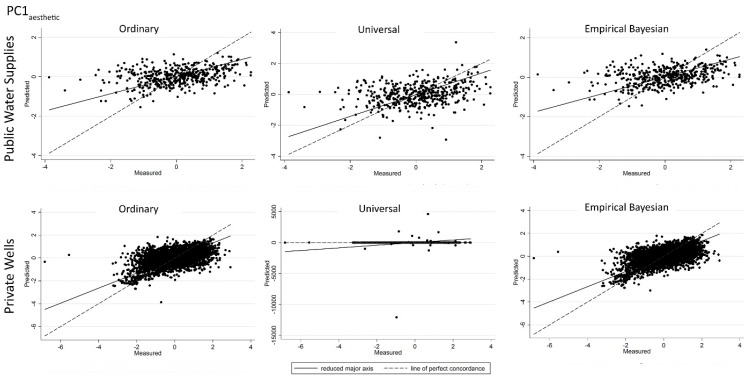
Predicted vs. measured values for PC1_aesthetic_ scores obtained from the cross-validation results for ordinary, universal, and empirical Bayesian kriging for: public water supplies (**top row**); and private wells (**bottom row**). Line of perfect concordance indicated by dashed line.

**Figure A6:**
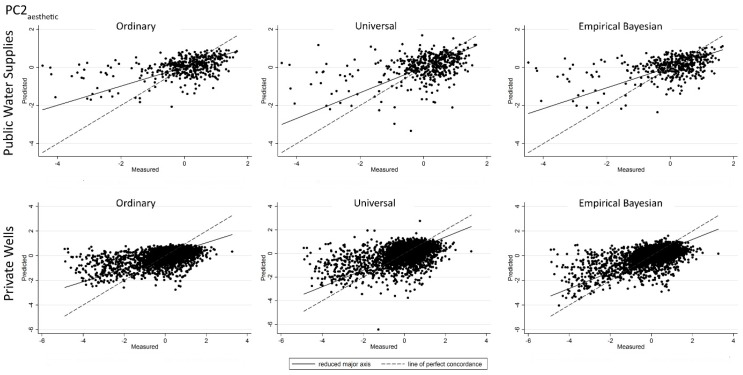
Predicted vs. measured values for PC2_aesthetic_ scores obtained from the cross-validation results for ordinary, universal, and empirical Bayesian kriging for: public water supplies (**top row**); and private wells (**bottom row**). Line of perfect concordance indicated by dashed line.

**Figure A7:**
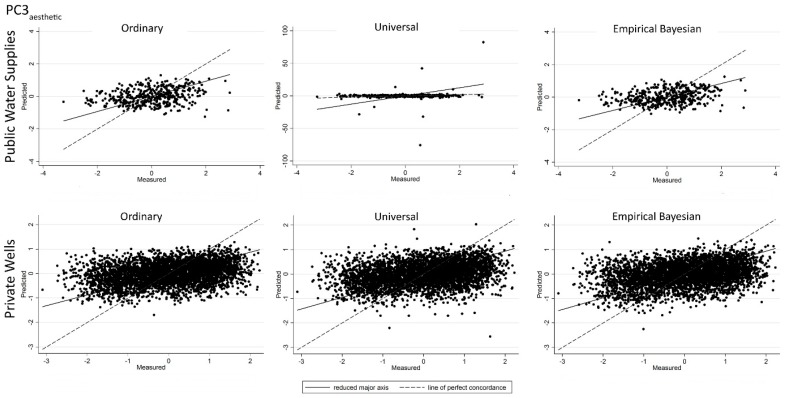
Predicted vs. measured values for PC3_aesthetic_ scores obtained from the cross-validation results for ordinary, universal, and empirical Bayesian kriging for: public water supplies (**top row**); and private wells (**bottom row**). Line of perfect concordance indicated by dashed line.

**Figure A8:**
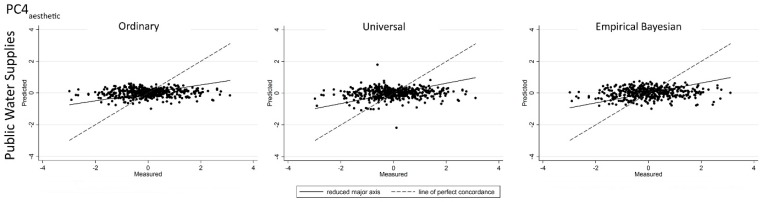
Predicted vs. measured values for PC4_aesthetic_ scores obtained from the cross-validation results for ordinary, universal, and empirical Bayesian kriging for public water supplies. Line of perfect concordance indicated by dashed line.

For each type of kriging, cross-validation plots of predicted vs. measured values (Appendix A, Figure A1, Figure A2, Figure A3, Figure A4, Figure A5, Figure A6, Figure A7 and Figure A8) revealed that, for low measured values, predicted values tended to be overestimated, and that, for higher measured values, predicted values tended to be underestimated.

**Figure 2:**
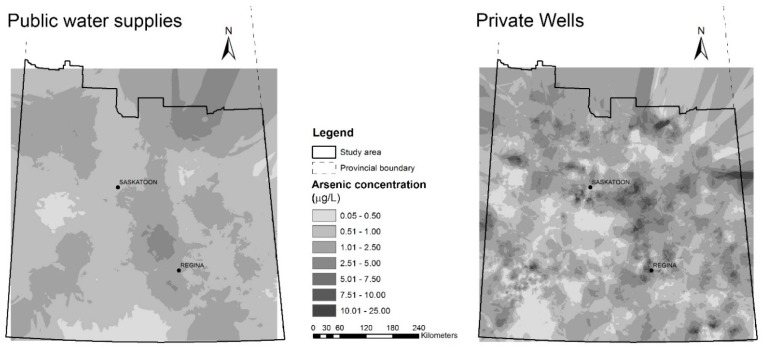
Arsenic concentrations (μg/L) predicted by empirical Bayesian kriging for: public water supply data (**left**); and private well data (**right**) in study area in southern Saskatchewan.

Results from empirical Bayesian kriging of log transformed arsenic concentrations from the public and private water supply data within the study area were back transformed to obtain surfaces of predicted arsenic concentrations (Figure 2). 

**Figure 3:**
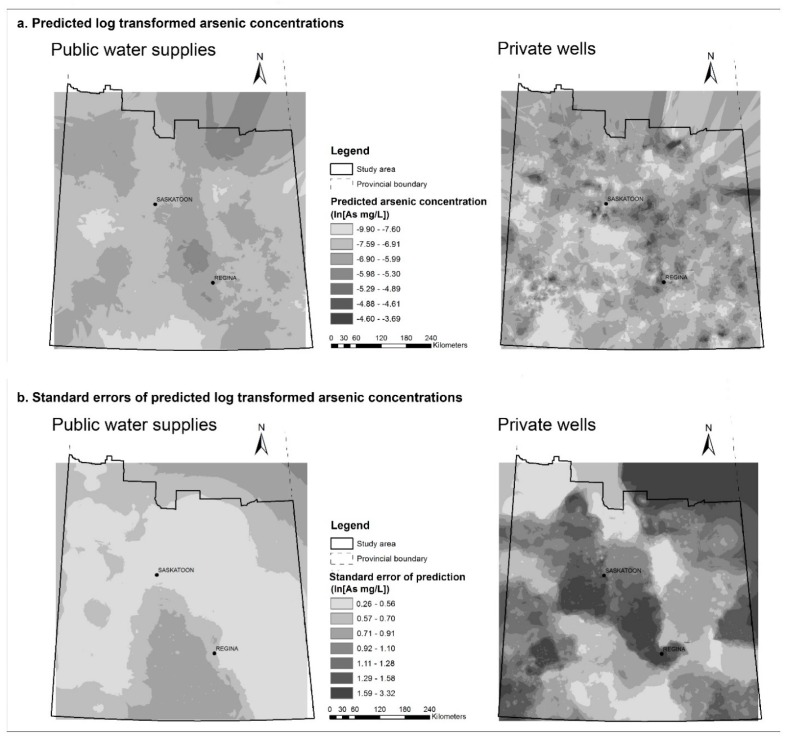
Empirical Bayesian kriging prediction maps for natural logarithm transformed arsenic concentrations (**a**); and the prediction standard error maps (**b**) for public supplies (left) and private supplies (right) in study area in southern Saskatchewan.

**Figure A9:**
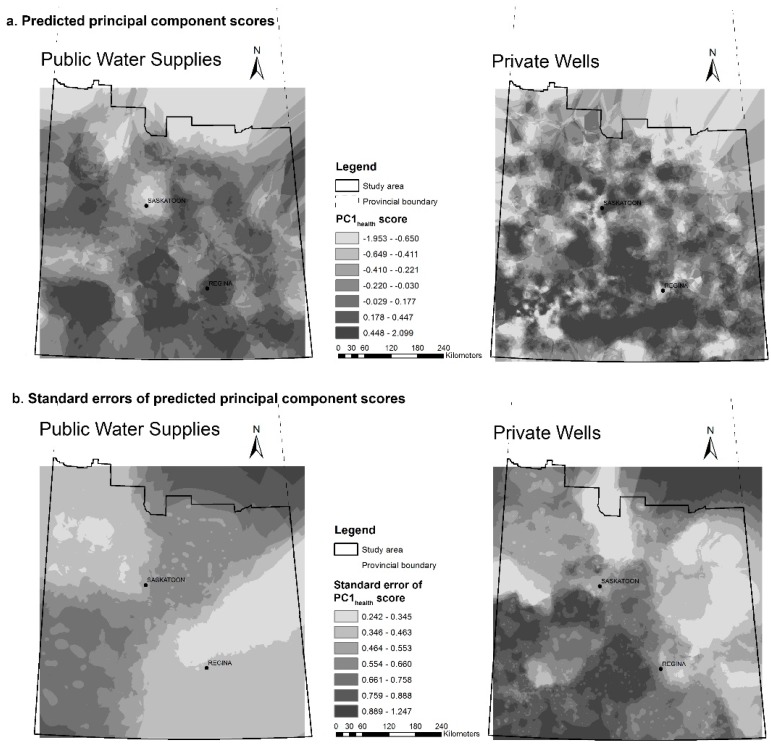
(**a**) Predicted PC1_health_ scores for public water supplies and private wells in study area; and (**b**) the corresponding prediction standard errors.

**Figure A10:**
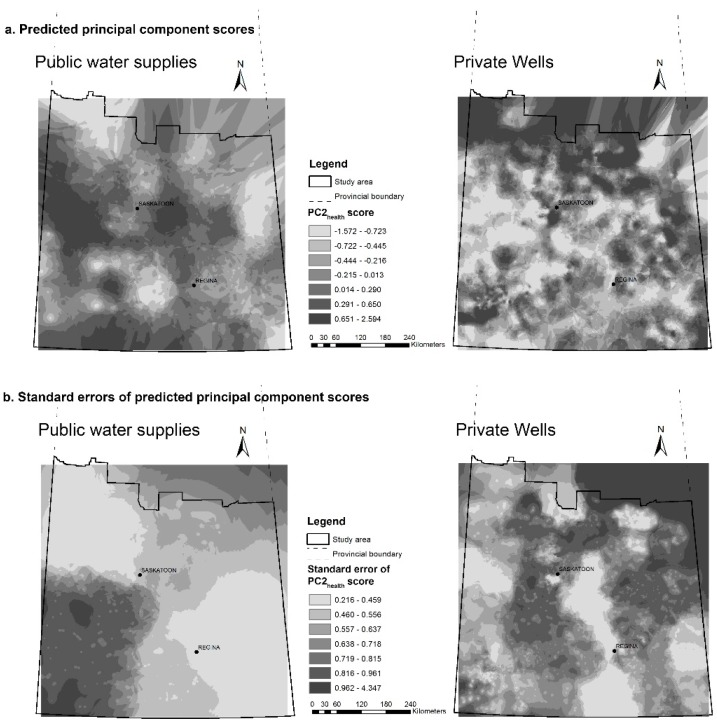
(**a**) Predicted PC2_health_ scores for public water supplies and private wells in study area; and (**b**) the corresponding prediction standard errors.

**Figure A11:**
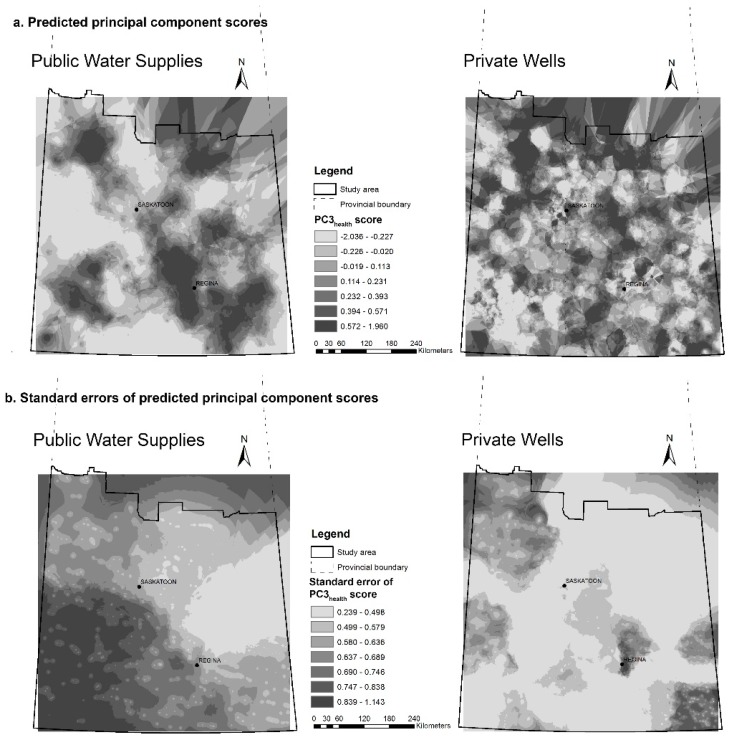
(**a**) Predicted PC3_health_ scores for public water supplies and private wells in study area; and (**b**) the corresponding prediction standard errors.

**Figure A12:**
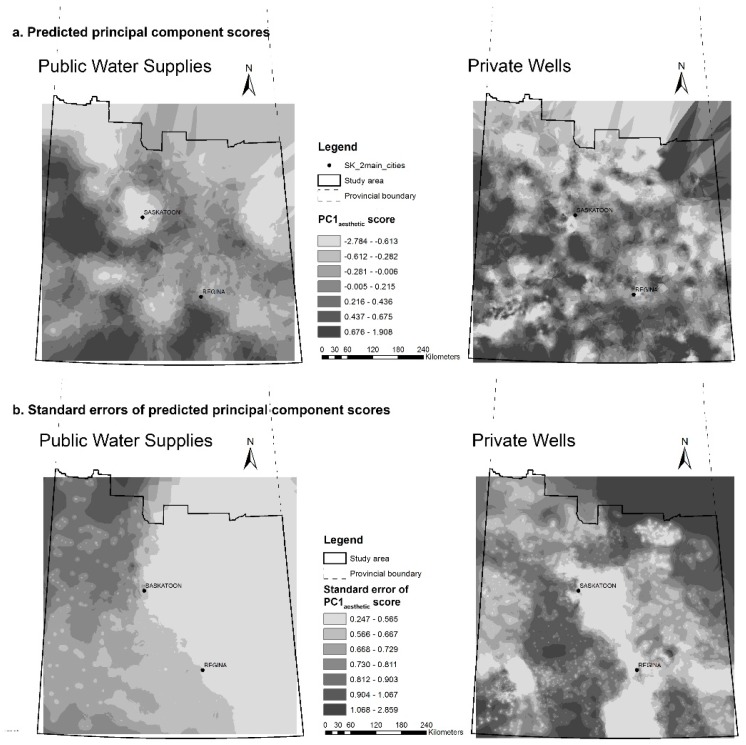
(**a**) Predicted PC1_aesthetic_ scores for public water supplies and private wells in study area; and (**b**) the corresponding prediction standard errors.

**Figure A13:**
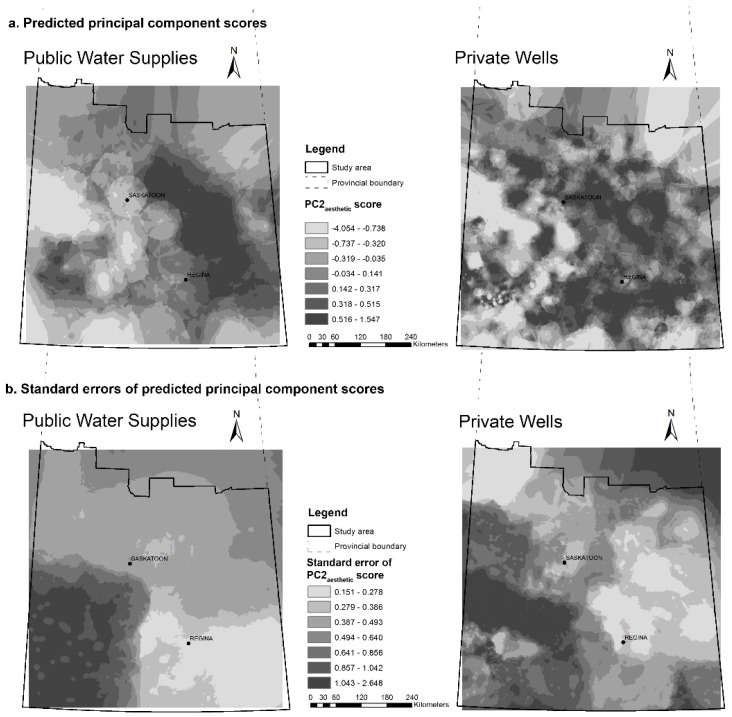
(**a**) Predicted PC2_aesthetic_ scores for public water supplies and private wells in study area; and (**b**) the corresponding prediction standard errors.

**Figure A14:**
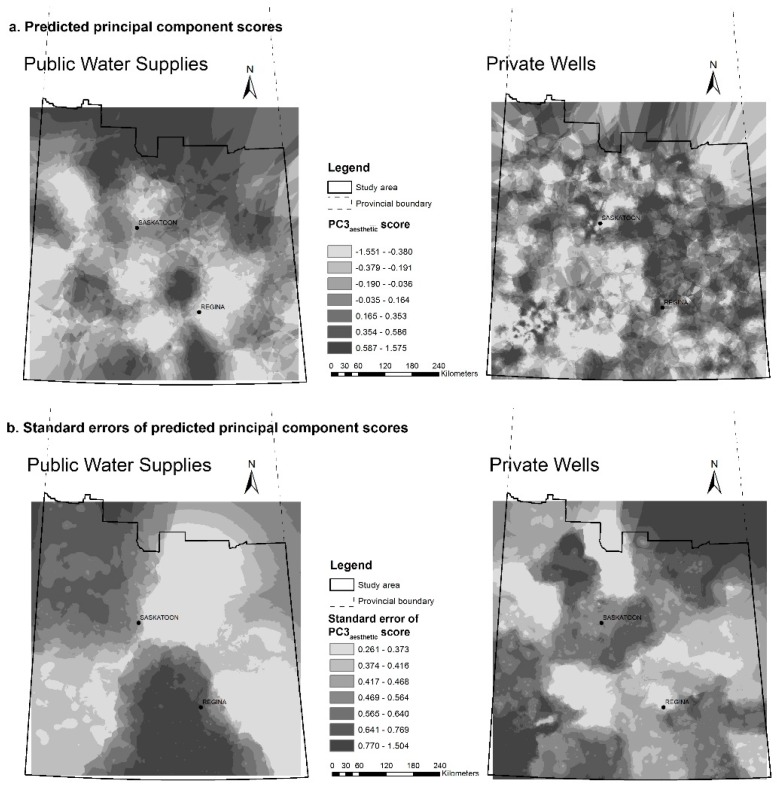
(**a**) Predicted PC3_aesthetic_ scores for public water supplies and private wells in study area; and (**b**) the corresponding prediction standard errors.

**Figure A15:**
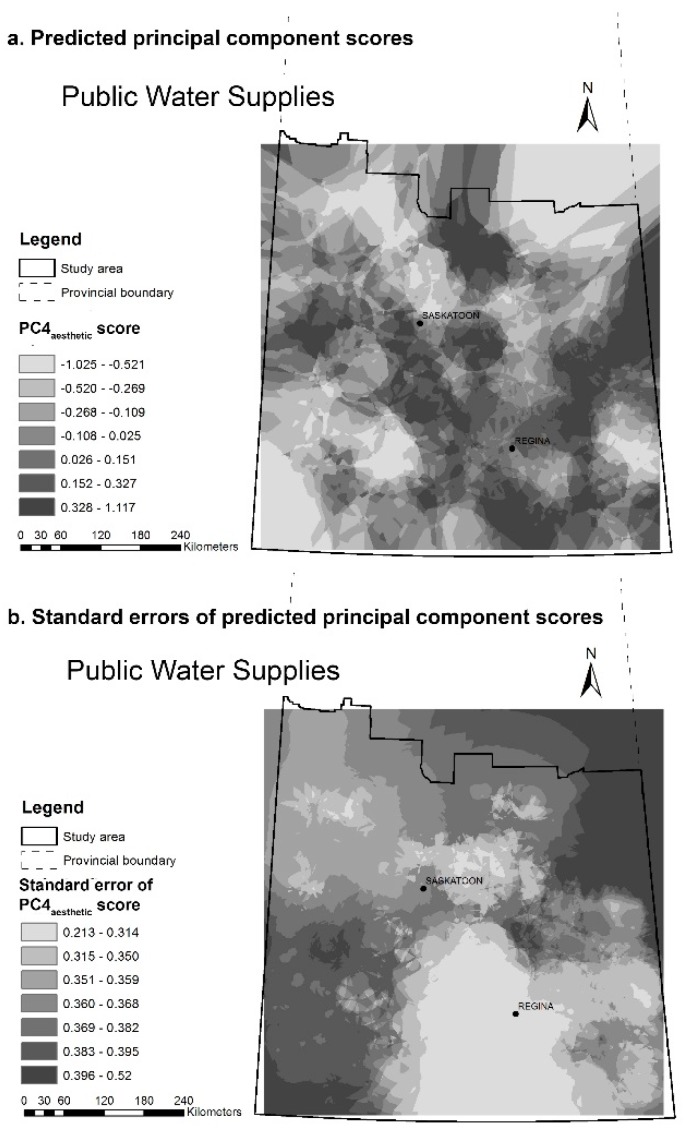
(**a**) Predicted PC4_aesthetic_ scores for public water supplies in study area; and (**b**) the corresponding prediction standard errors. The fourth principal component was extracted for public water supply data only.

Prediction surfaces based on the natural logarithm transformed arsenic concentrations are shown in Figure 3a, along with their corresponding prediction standard error surfaces, which illustrate the amount of uncertainty associated with the predicted values (Figure 3b). Surfaces for each of the principal components are available in Appendix B (Figure A9, Figure A10, Figure A11, Figure A12, Figure A13, Figure A14 and Figure A15).

## 4. Discussion

In Saskatchewan, groundwater sources are primarily utilized by residents of rural and remote areas [53]. A considerable number of water samples exceeded drinking water standards and objectives in the surveillance data, highlighting the need to promote adequate testing of drinking water in rural areas. Generally, it appears that contaminants listed as aesthetic objectives exceed guideline values at a higher frequency than the health-related standards. As expected, the raw groundwater sampled from private wells exceeded standards and objectives more frequently than the water from the regulated, treated public water supplies. However, a considerable number of samples from public supplies still exceeded guidelines, especially for aesthetic objectives.

In a previous study, a sample of 283 wells in Saskatchewan [19] found that approximately 45% of the wells exceeded the Saskatchewan drinking water objective for sulfate, 47% exceeded the objective for iron, 61% exceeded the objective for hardness, and 79% exceeded the objective for manganese. Our study reflected a similar pattern, although the rates of exceedances were slightly lower; approximately 39% for sulfate, 40% for iron, 31% for hardness, and 68% for manganese in the private wells, and 32% for sulfate, 19% for iron, 21% for hardness and 53% for manganese in public supplies. A previous study from Saskatchewan reported that having aesthetic complaints about tap water was associated with the perception that tap water was unsafe [24]. The aesthetic quality of tap water could act as a determinant of health by increasing consumption of water alternatives which may include sugar-sweetened beverages [8].

Previous studies have also investigated concentrations of arsenic [17] and nitrate [18] in Saskatchewan wells. Thompson et al. [17] sampled 61 wells (private wells and wells maintained by rural municipalities) for arsenic, and found that 23% exceeded the current Saskatchewan drinking water standard applied to regulated public water supplies. In our study, just over 13% of private wells exceeded the standard, while approximately 7% of public supply samples exceeded the standard. Thompson [18] found that 14% of wells tested exceeded the standard for nitrate for regulated waterworks, while 12% of the private wells included in our study exceeded the standard. However, only 4% of public supplies exceeded the nitrate standard. 

### 4.1. Principal Components Analysis

PCA has been used in previous studies to examine and interpret patterns of groundwater quality parameters [30,31,32,33,34,54]. These types of studies typically identify common factor patterns and interpret them with respect to presumed natural and anthropogenic processes that impact groundwater quality, and are often focused on major ions (e.g., sodium, chloride, magnesium, sulfate) that would fall under aesthetic objectives in the Saskatchewan Drinking Water Quality Standards and Objectives. PCA analysis of groundwater has often included nitrate, which falls under Saskatchewan health standards, as a marker for anthropogenic influences on groundwater (e.g., [30,31,32,34]). However, the full range of parameters included in such studies has not been consistent, particularly with respect to the inclusion of trace metals, making it somewhat difficult to compare results. Comparison to our study was further hampered because we analyzed health standards and aesthetic objectives separately to align our analysis with Saskatchewan Drinking Water Quality Standards and Objectives.

We limited our analyses to include parameters that were routinely sampled from both the public and private supply data to facilitate comparison between the differing supplies. We expected the results to differ between the types of systems because the public supply data represent treated water supplies and the private well data represent raw water samples. While there were some differences in the principal components extracted from the public and private data, there were some striking similarities, especially in the results for the aesthetic objectives, even though four PC_aesthetic_ were retained for the public supply data and three for the private well data. The first PC_aesthetic_ was associated with the same group of variables in both datasets: sodium, chloride, sulfate, alkalinity and total dissolved solids. Additionally, hardness and magnesium were strongly associated with the second PC, and iron and manganese with the third PC in both public and private water supplies. The consistent patterns of these parameters between the datasets suggest relatively strong associations between these parameters in Saskatchewan groundwater.

The PCA for health standards also exhibited some consistencies: nitrate and selenium were strongly associated with the first PC1_health_ for both public and private supplies. Arsenic was associated with the third PC_health_ in both datasets, but strongly associated with that PC in the public supply data. In contrast, uranium was associated with PC3_health_ in public supplies and with PC1_health_ in private wells. In addition, lead was associated with PC1_health_ in public supplies, and with PC3_health_ in private wells. Because lead contamination of water can be associated with leaching from distribution systems, differences in the covariance of lead with other parameters between public supplies and private wells is not unexpected. However, caution is warranted in the interpretation of the PCA for health standards from the public supply data considering the low Kaiser’s measure of sampling adequacy for these data.

### 4.2. Geostatistical Analysis

Kriging has previously been validated as a method to summarize arsenic concentrations in groundwater quality and in one study was found to be superior to using an area average or nearest well as a proxy to predict well concentrations [26]. While some studies have investigated the use of indicator kriging to model the probability of higher arsenic concentrations using geological and hydrological covariates [25,27], some recent studies have compared various kriging methods that are accessible in GIS software to investigate prediction of arsenic concentrations in groundwater [28,29]. James et al. [29] evaluated the performance of various kriging methods (ordinary, universal, simple kriging with varying means, kriging with external drift, cokriging with ordinary kriging and cokriging with universal kriging) over a relatively small area in Colorado and found that ordinary kriging performed best. Gong et al. [28] compared inverse distance weighted interpolation with kriging using Gaussian and spherical models as well as cokriging in predicting arsenic concentrations over various regions in Texas, and found regional differences in the performance of kriging, and concluded that kriging over smaller areas was more accurate than over large geographic regions.

In the present study, Bayesian kriging had the lowest RMSE for the greatest number of variables and was considered the optimal method for our data. However, values of RMSE for ordinary kriging were very similar to those for Bayesian kriging, so there does not appear to be much difference between these methods in the accuracy of the predicted values on cross validation. 

We elected not to use covariate information such as well depths or geological data in our models due to difficulty in obtaining accurate covariate data over our study area. Furthermore, a previous study in SK demonstrated a lack of correlation between well depths and concentrations of the water parameters studied [55]. While depth might be expected to improve modeling of arsenic concentration, conflicting results from other studies suggest that the contribution of depth may be dependent on the study area. For example, a negative correlation between increasing well depth and arsenic concentrations has been reported for wells in Bangladesh [56,57], while a positive association between well depth and arsenic was reported in North Carolina [58]. Yang et al. [27] did not detect any association between arsenic concentration and well depth in Maine. In one study, including well depth in cokriging models did not improve the ability of kriging to predict arsenic levels [29]. Gong et al. [28] found that incorporating well depth in cokriging did not necessarily improve the correlation between predicted and actual values, but did improve the performance of regression models used to predict arsenic levels. Furthermore, Yu et al. [57] investigated factors affecting arsenic at different geographic scales and concluded that much of the variability in arsenic concentrations at a scale of less than 3 km could be explained by well depth, while geology was the most important factor at scales of greater than 10 km. This suggests that given the large scale of our study area relative to other reported studies, it is unlikely that adding well depth as a covariate would have improved our models. While incorporation of geological data might have improved our predictions, this information was not available for the large study area.

Others have reported a tremendous amount of heterogeneity in groundwater concentrations of arsenic over small scales that is poorly understood [27,57]. In Bangladesh, wells within a radius of less than 1 km were found to vary by up to 1000 μg/L [57]. In another study of a relatively small region of Bangladesh, wells in close proximity exhibited extremely variable arsenic concentrations, especially wells less than 30 m in depth [59]. This issue was also highlighted in the geostatistical analysis of arsenic in wells in Michigan; residuals for predicted arsenic values were mapped and no spatial pattern in the residuals was detected [26]. The close proximity of wells with negative and positive residuals of greater than 10 μg/L reflected high variability in arsenic concentrations over short distances [26]. Additionally, a study in Texas compared geostatistical methods among regions, and found the performance of the different methods varied less within a given area than across the different regions [28]. This suggests that variability in the distribution of groundwater arsenic across regions is a limiting factor in identifying a single method that would perform uniformly well in different geographic areas. Given the apparent differences in processes influencing spatial variability of arsenic at different scales, it is possible that developing kriging models over smaller targeted areas with a high density of samples could improve the performance of predictions for some local regions. However, this analysis was intended to estimate the mean arsenic concentrations along with principal components representing drinking water quality over a total area of approximately 327,900 km^2^.

Interpretation of mapped results of PCA is less straightforward because the values are a representation of a combination of parameters that contribute to the PCA components. For example, areas with high values for the first PC for aesthetic objectives represent higher predicted concentrations of one or more of the contributors to this component, including sodium, chloride, sulfate, alkalinity and total dissolved solids. Nevertheless, this method is useful for examining patterns in common grouping of parameters and allowed extraction of factor scores to summarize mixtures of variables over geographic regions for use in epidemiological analyses.

Previous studies have used geostatistical methods to map the scores resulting from PCA or FA and used the resultant maps to predict the factors that may be impacting groundwater quality, such as pollution or salt water intrusion [34,35,36,37]. It does not appear that the use of kriging with PCA or factor analysis has been well-validated for prediction of groundwater quality. We are not aware of other studies that have assessed the ability of kriging to accurately predict PCA scores at unmeasured locations so we have not compared our results to others.

We would expect to see a reduction in predictive ability by combining a variable reduction method such as PCA with kriging. PCA reduces the dimensionality of a dataset while capturing as much of the information in the original variables as possible. In our data, the percentage of variance in the original measures described by the retained PCs ranged 63.7–77.8%. While spatial patterns of arsenic have been studied extensively, spatial patterns of the other variables, and especially mixtures of variables, have not. Therefore, it is possible that the PCs we extracted are subject to variability at scales not captured by our analysis. The use of PCA combined with kriging of factor scores should not be discounted as a means of summarizing water quality but should be investigated further, ideally with higher density sampling over smaller geographical regions.

Although the data from public water supplies consisted of repeated measures over time for most sites, the decision was made to model a mean value for each parameter at each site rather than specifically estimating concentrations and component scores at particular points in time. The capacity to estimate time-specific values was limited as not all sites had the same intensity of sampling and sufficient samples to provide a precise estimate for every year in the dataset. Finally, and most importantly, the primary objective of this study was to estimate exposures to metals and ions in drinking water for an epidemiologic study of associations between water quality and chronic diseases with uncertain induction periods. The relevant exposure period over which environmental exposures contribute to chronic diseases is uncertain and represents a potential source of misclassification [60,61]. Because the precise time period of interest for estimating exposure was unknown, an estimate of average past exposure was deemed more appropriate for the planned epidemiological analysis than estimating exposures for specific time points, for example, using space-time kriging models.

### 4.3. Limitations

It is estimated that there are over 66,000 wells in Saskatchewan [17] and our sample of 4093 private wells is a non-random sample of less than 10% of privately owned wells in the province. Because the database consists of samples taken through participation in a voluntary water quality program, it could disproportionately represent residents with concerns about their well water quality. The results from private wells and public water supplies were similar, suggesting this was not a substantial issue. 

Although the public supply data represent data from all available public water supplies across Saskatchewan, there were relatively few locations represented in the public supply data relative to the size of the study area, resulting in a low sampling density that may have particularly impacted the ability of kriging to capture the variability of arsenic at small spatial scales. 

Our PCA may have been hampered by not being able to make use of a full suite of parameters and high proportions of concentrations below detection limits for some variables, especially with respect to the health-related standards. We also made the decision to separately analyze aesthetic and health parameters because they are segregated into drinking water standards and objectives. It is possible that considering all available parameters together could have improved the performance of the PCA, although it seems likely that the high number of samples below detection limits would continue to limit the usefulness of some of the variables measured as health standards. 

Kriging methods rely on an estimation of the spatial structure of data. While semivariogram models provide a means of investigating spatial relationships, kriging typically requires the assumption that the chosen semivariogram model represents the true spatial structure. This assumption is relaxed with empirical Bayesian kriging allowing for uncertainty in the semivariogram parameters which likely contributes to the superior predictive performance of this method in our study. However, the performance of empirical Bayesian kriging was only marginally better than that of ordinary kriging. Other researchers have investigated Bayesian statistical methods to predict arsenic groundwater concentrations which incorporate spatial relationships using alternatives to semivariograms [58,62]. Methods such as these could potentially be used to improve prediction of arsenic concentrations and overcome some of the limitations of kriging especially when spatial variability arises from processes at different scales, limiting the effectiveness of variogram modeling even after allowing for uncertainty in the semivariogram.

## 5. Conclusions

In this study, we investigated the use of PCA and kriging to predict groundwater water quality across southern Saskatchewan as part of the exposure assessment for epidemiological investigation of links between water quality and chronic disease.

A considerable number of samples from rural public water supplies and private wells exceeded the standards and objectives set by the province of SK for regulated supplies, highlighting the need for study of potential health impacts associated with consumption of drinking water of poor quality in rural populations. Arsenic concentrations exceeded the health standard of 0.01 mg/L in 7% of public water supplies and 13% of private wells. Guidelines for aesthetic objectives were exceeded with greater frequency than health standards. These results suggest that the investigation of health impacts of unpalatable water and mixtures of contaminants may be particularly important.

We compared ordinary, universal and Bayesian kriging for predicting log arsenic concentrations and PC scores across the study area for public and private water supplies. Across the variables investigated, empirical Bayesian kriging resulted in the greatest accuracy of the most predicted values.

As a method that is accessible in commercial software packages, empirical Bayesian kriging represents a flexible and viable statistical technique that could be used to summarize water quality as a method of exposure assessment. While geostatistical analysis may be more informative if applied over smaller regions and higher sampling densities, the objective of this study was to estimate exposure to drinking water parameters over a very large geographic area in rural Saskatchewan.

## Figures and Tables

**Table 1:** Descriptive statistics for individual samples in public water supplies in Saskatchewan during 1985–2012, along with the Saskatchewan standards and objectives, percent of samples exceeding the respective standard or objective, and percent of samples below detection limit for the water parameters included in the analysis.

	Samples	Mean	SD	Median	P95	Max	SK Standard	Exceedances	Below DL
	(n)	(mg/L)	(mg/L)	(mg/L)	(mg/L)	(mg/L)	(mg/L)	Percent of Samples	Percent of Samples
***Health standards***									
Arsenic	4732	0.003	0.007	0.001	0.014	0.098	0.01	6.9	22.9
Barium	4485	0.05	0.10	0.02	0.18	2.40	1	0.04	2.8
Boron	4116	0.36	0.50	0.24	1.20	6.00	5	0.2	5.5
Lead	4569	0.0019	0.0078	0.0005	0.0070	0.4100	0.01	2.5	67.3
Nitrate	9562	11.6	20.5	3.0	42.0	933.0	45	4.1	31.4
Selenium	4527	0.001	0.006	0.001	0.004	0.140	0.01	1.9	72.2
Uranium	4617	0.006	0.011	0.003	0.023	0.180	0.02	7.3	16.9
***Aesthetic objectives***							**SK Objective**		
Alkalinity	5404	408	154	408	674	2451	500	22.0	0
Chloride	5435	48.5	86.9	18.0	233.4	1803.0	250	4.2	3.8
Copper	4497	0.084	0.290	0.018	0.310	6.200	1	1.2	6.8
Hardness	4162	536	341	489	1107	7800	800	20.7	0.2
Iron	4587	0.30	1.33	0.06	1.08	46.00	0.3	18.8	5.9
Magnesium	3120	55.5	37.4	49.0	125.0	449.0	200	0.002	4.3
Manganese	4614	0.26	1.58	0.07	0.98	101.00	0.05	53.5	7.2
Sodium	4353	162	190	80	585	1868	300	18.8	0.1
Sulfate	4284	403	367	326	1045	9000	500	32.5	2.5
TDS	4290	1283	661	1199	2453	6687	1500	34.6	0
Zinc	4481	0.03	0.24	0.01	0.08	11.00	5	0.04	25.4

SD = standard deviation, P95 = 95th percentile, max = maximum, DL = detection limit, TDS = Total Dissolved Solids.

**Table 2:** Descriptive statistics for individual samples in private water supplies in Saskatchewan during 1996–2011, along with the Saskatchewan standards and objectives, percent of samples exceeding the respective standard or objective, and percent of samples below detection limit for the water parameters included in the analysis.

	Samples	Mean	SD	Median	P95	Max	SK Standard	Exceedances	Below DL
	(n)	(mg/L)	(mg/L)	(mg/L)	(mg/L)	(mg/L)	(mg/L)	Percent of Samples	Percent of Samples
***Health Standards***									
Arsenic	4082	0.005	0.012	0.001	0.023	0.210	0.01	13.5	21.3
Barium	4082	0.08	0.14	0.03	0.26	2.19	1	0.4	0.3
Boron	4082	0.33	0.54	0.15	1.40	7.10	5	0.2	1.8
Lead	4082	0.0007	0.0043	0.0005	0.0014	0.2100	0.01	0.7	72.9
Nitrate	3996	24.5	73.5	1.20	126.0	1300.0	45	12.2	27.4
Selenium	4076	0.008	0.036	0.001	0.033	0.840	0.01	11.2	41.1
Uranium	4076	0.012	0.021	0.005	0.044	0.400	0.02	17.8	11.9
***Aesthetic Objectives***							**SK Objective**		
Alkalinity	4019	416	148	399	671	1620	500	21.8	n/a
Chloride	4019	69.8	178.0	21.0	257.0	4090.0	250	5.2	1.7
Copper	4080	0.011	0.037	0.003	0.044	1.100	1	0.02	24.1
Hardness	4019	695	569	557	1760	6810	800	30.7	0.3
Iron	4091	1.24	2.81	0.12	6.00	40.00	0.3	40.5	1.2
Magnesium	4019	81.0	84.4	60.0	220.0	1450.0	200	6.1	0.3
Manganese	4091	0.44	0.68	0.18	1.70	11.00	0.05	68.2	2.5
Sodium	4019	181	237	84	653	2710	300	20.8	0
Sulfate	4019	546	618	354	1680	7690	500	39.1	0.15
TDS	4019	1560	1030	1330	3450	11300	1500	42.7	n/a
Zinc	4081	0.19	1.00	0.02	0.76	31.00	5	0.4	15.8

SD = standard deviation, P95 = 95th percentile, max = maximum, DL = detection limit, n/a = no detection limit available, TDS = Total Dissolved Solids.

**Table 3:** Descriptive statistics for predicted concentrations after repeated measures modeling for each parameter by site for public water supplies in Saskatchewan, along with Saskatchewan standards and objectives, and the percent of sites with a predicted concentration exceeding the standard or objective. A summary of the number of samples per site incorporated in repeated measures models for each parameter is also shown.

		Mean Predicted Concentration							Number of Samples per Site		
	Sites	Mean	SD	Median	P95	Max	SK Standard	Exceed	Min	Median	Max
	(n)	(mg/L)	(mg/L)	(mg/L)	(mg/L)	(mg/L)	(mg/L)	Percent of Sites	(n)	(n)	(n)
***Health Standards***											
Arsenic	492	0.002	0.003	0.001	0.007	0.039	0.01	2.0	1	9	59
Barium	491	0.04	0.07	0.02	0.13	0.74	1	0	1	9	28
Boron	477	0.35	0.41	0.23	1.13	3.03	5	0	1	9	26
Lead	491	0.0006	0.00004	0.0006	0.0007	0.0007	0.01	0	1	9	28
Nitrate	497	2.67	6.24	1.03	10.42	95.18	45	0.2	1	11	366
Selenium	492	0.001	0.001	0.0003	0.002	0.012	0.01	0.2	1	9	28
Uranium	491	0.005	0.006	0.003	0.016	0.076	0.02	2.6	1	9	33
***Aesthetic Objectives***							**SK Objective**				
Alkalinity	503	400	127	400	612	900	500	18.1	1	10	36
Chloride	499	42.9	64.2	19.8	173.7	489.8	250	2.4	1	10	257
Copper	492	0.024	0.025	0.015	0.075	0.170	1	0	1	9	28
Hardness	501	492	296	457	10497	14827	800	15.2	1	8	32
Iron	482	0.10	0.12	0.060	0.29	1.18	0.3	4.1	1	9	34
Magnesium	483	52.3	34.0	46.4	116.1	191.9	200	0	1	6	26
Manganese	483	0.12	0.19	0.06	0.45	1.84	0.05	52.6	1	9	34
Sodium	488	160	176	87	561	883	300	18.0	1	8	34
Sulfate	480	383	318	304	995	1930	500	30.0	1	8	34
TDS	487	1277	590	1189	2355	3467	1500	33.1	1	8	34
Zinc	491	0.01	0.01	0.01	0.02	0.10	5	0	1	9	28

SD = standard deviation, Min = minimum, P95 = 95th percentile, Max = maximum, Exceed = predicted concentration exceeds standard or objective, TDS = Total Dissolved Solids.

**Table 4:** Principal components analysis on public water supplies and private well water supplies: varimax rotated principal components patterns, eigenvalues and percent of variance explained for each retained component. The loadings in bold font indicate the principal component with the maximum loading for each measured variable.

	Public Water Supplies				Private Wells		
***Health Standards***							
	PC1	PC2	PC3		PC1	PC2	PC3
Arsenic	−0.121	0.142	**0.808**		−0.341	−0.091	**0.474**
Barium	0.047	−**0.818**	−0.141		−0.041	**0.893**	0.100
Boron	−0.062	**0.903**	−0.123		−0.195	−**0.818**	0.168
Lead	**0.472**	0.092	0.171		0.156	0.026	**0.893**
Nitrate	**0.768**	−0.071	−0.164		**0.770**	0.275	−0.110
Selenium	**0.867**	−0.220	0.019		**0.853**	−0.007	0.074
Uranium	0.387	−0.290	**0.576**		**0.772**	−0.013	−0.049
Eigenvalue	2.127	1.275	1.059		2.290	1.381	1.057
Cumulative variance (%)	30.4	48.6	63.7		32.7	52.5	67.6
***Aesthetic Objectives***							
	PC1	PC2	PC3	PC4	PC1	PC2	PC3
Alkalinity	**0.755**	0.111	0.164	−0.198	**0.687**	−0.023	0.217
Chloride	**0.753**	−0.193	0.002	0.226	**0.779**	0.043	−0.195
Copper	0.127	0.012	−0.200	**0.714**	0.030	0.223	−**0.757**
Hardness	0.009	**0.973**	0.066	0.042	0.067	**0.960**	0.038
Iron	0.138	−0.089	**0.901**	0.053	0.117	0.121	**0.784**
Magnesium	−0.014	**0.961**	0.055	0.038	0.103	**0.951**	0.020
Manganese	0.188	0.452	**0.711**	−0.065	0.062	0.468	**0.663**
Sodium	**0.914**	−0.199	0.136	0.026	**0.922**	−0.116	0.118
Sulfate	**0.663**	0.517	0.018	0.116	**0.609**	0.555	0.076
Total Dissolved Solids	**0.920**	0.288	0.121	−0.016	**0.907**	0.325	0.082
Zinc	−0.089	0.078	0.237	**0.763**	−0.091	**0.396**	−0.375
Eigenvalue	3.746	2.362	1.264	1.181	3.775	2.184	1.779
Cumulative variance (%)	34.1	55.5	67.0	77.8	34.3	54.2	70.4

PC = principal component.

**Table 5:** Summary of parameters used as input for variogram models for ordinary and universal kriging.

Variable	Number of Sites	Lag Distance (km)	Number of Lags	Large Scale Trend
*Public Supplies*				
Arsenic	480	13.03	31	2nd order
PC1_health_	459	13.44	30	2nd order
PC2_health_	459	13.44	30	2nd order
PC3_health_	459	13.44	30	2nd order
PC1_aesthetic_	435	13.84	29	2nd order
PC2_aesthetic_	435	13.84	29	2nd order
PC3_aesthetic_	435	13.84	29	2nd order
PC4_aesthetic_	435	13.84	29	1st order
*Private Supplies*				
Arsenic	4073	2.52	100	1st order
PC1_health_	3970	2.55	100	2nd order
PC2_health_	3970	2.55	100	2nd order
PC3_health_	3970	2.55	100	2nd order
PC1_aesthetic_	3999	2.54	100	2nd order
PC2_aesthetic_	3999	2.54	100	2nd order
PC3_aesthetic_	3999	2.54	100	1st order

PC = principal component.

**Table 6:** Root mean square error values for each parameter obtained from cross validation analysis of ordinary, universal and empirical Bayesian kriging models for public water supply and private well data. The lowest RMSE for each variable is bolded.

	Ordinary	Universal	Bayesian
*Municipal Systems*			
Arsenic	**1.0115**	1.2634	1.0180
PC1_health_	**0.9730**	1.0300	0.9734
PC2_health_	**0.9510**	3.5363	0.9586
PC3_health_	0.8987	1.0865	**0.8925**
PC1_aesthetic_	0.9276	1.0293	**0.9268**
PC2_aesthetic_	**0.8899**	0.9567	0.9013
PC3_aesthetic_	0.9993	6.2933	**0.9822**
PC4_aesthetic_	**1.0204**	1.0239	1.0232
*Private Wells*			
Arsenic	1.5949	1.6081	**1.5593**
PC1_health_	0.9130	212.78	**0.9090**
PC2_health_	0.8388	0.9111	**0.8200**
PC3_health_	**0.9536**	1.0299	0.9626
PC1_aesthetic_	0.8234	211.46	**0.8091**
PC2_aesthetic_	0.8618	0.8918	**0.8020**
PC3_aesthetic_	0.9630	0.9724	**0.9606**
